# Keratotic Basal Cell Carcinoma With Matrical Differentiation on the Flank: A Case Report

**DOI:** 10.7759/cureus.96222

**Published:** 2025-11-06

**Authors:** Vivek Vemugunta, Douglas Robins

**Affiliations:** 1 Dermatology, University of Central Florida College of Medicine, Orlando, USA; 2 Dermatology, University of Florida College of Medicine, Gainesville, USA

**Keywords:** case report, immunohistochemical staining (berep4), keratotic basal cell carcinoma, matrical differentiation, surgical excision

## Abstract

Basal cell carcinoma with matrical differentiation (BCCMD) is a rare histologic variant of basal cell carcinoma. We report a case of keratotic BCCMD on the right flank of a 57‑year‑old female, presenting as a 10 cm × 6 cm lesion that rapidly enlarged over the course of one year. Histopathology revealed basaloid lobules with central keratinization, resembling early pilar structures, a key feature of matrical differentiation. Immunohistochemical staining with BerEP4 confirmed the diagnosis. MRI demonstrated no deep or regional extension. The lesion was excised surgically and healed by secondary intent. This case is notable due to the lesion’s aggressive clinical growth and uncommon anatomic location. Recognizing matrical features is essential for accurate diagnosis, especially given the overlap with adnexal tumors such as pilomatricoma and pilomatrix carcinoma. Although BCCMD rarely metastasizes, its potential for local destruction underscores the importance of early recognition and appropriate management.

## Introduction

Basal cell carcinoma (BCC) is the most common type of skin cancer, typically presenting in subtypes such as nodular, superficial, or infiltrative [1]. Approximately 80% of skin cancer cases have been attributed to BCC, and it affects 3.3 million Americans annually and requires substantial resources to treat [1,2]. Typically, BCCs affect adults with lighter phenotypes [3]. However, BCC with matrical differentiation (BCCMD) is an exceedingly rare variant, characterized by features reminiscent of hair matrix cells, such as islands of shadow cells [4]. As of 2018, only 42 cases have been detailed in the literature, underscoring its rarity [4,5]. Since then, only a few additional cases have been reported, which have limited clinical familiarity and contributed to diagnostic uncertainty.

This uncommon histopathological presentation can pose diagnostic challenges, particularly in distinguishing it from other adnexal tumors or cutaneous malignancies with similar features. This tumor should be differentially diagnosed from other tumors, particularly a pilomatricoma and pilomatrix carcinoma [6]. These variants have key structural differences, including ghost cells and calcification in pilomatricomas, which help distinguish them from BCCMD. Furthermore, while BCC is generally known for its slow progression, this case is significant due to the aggressive nature of the tumor’s regional growth and spread [7]. Such unique behavior further complicates diagnosis and emphasizes the need for prompt recognition and intervention.

To contextualize this case, a review of previously reported cases is summarized, highlighting the clinical variability of this rare variant. While many cases involved facial lesions with indolent growth patterns, some displayed rapid enlargement or occurred in immunosuppressed patients, as seen in a renal-transplant patient. Our case is unique in that it presented on the trunk with rapid growth, reaching 10 cm × 6 cm within a year in an immunocompetent individual.

## Case presentation

**Figure 1:**
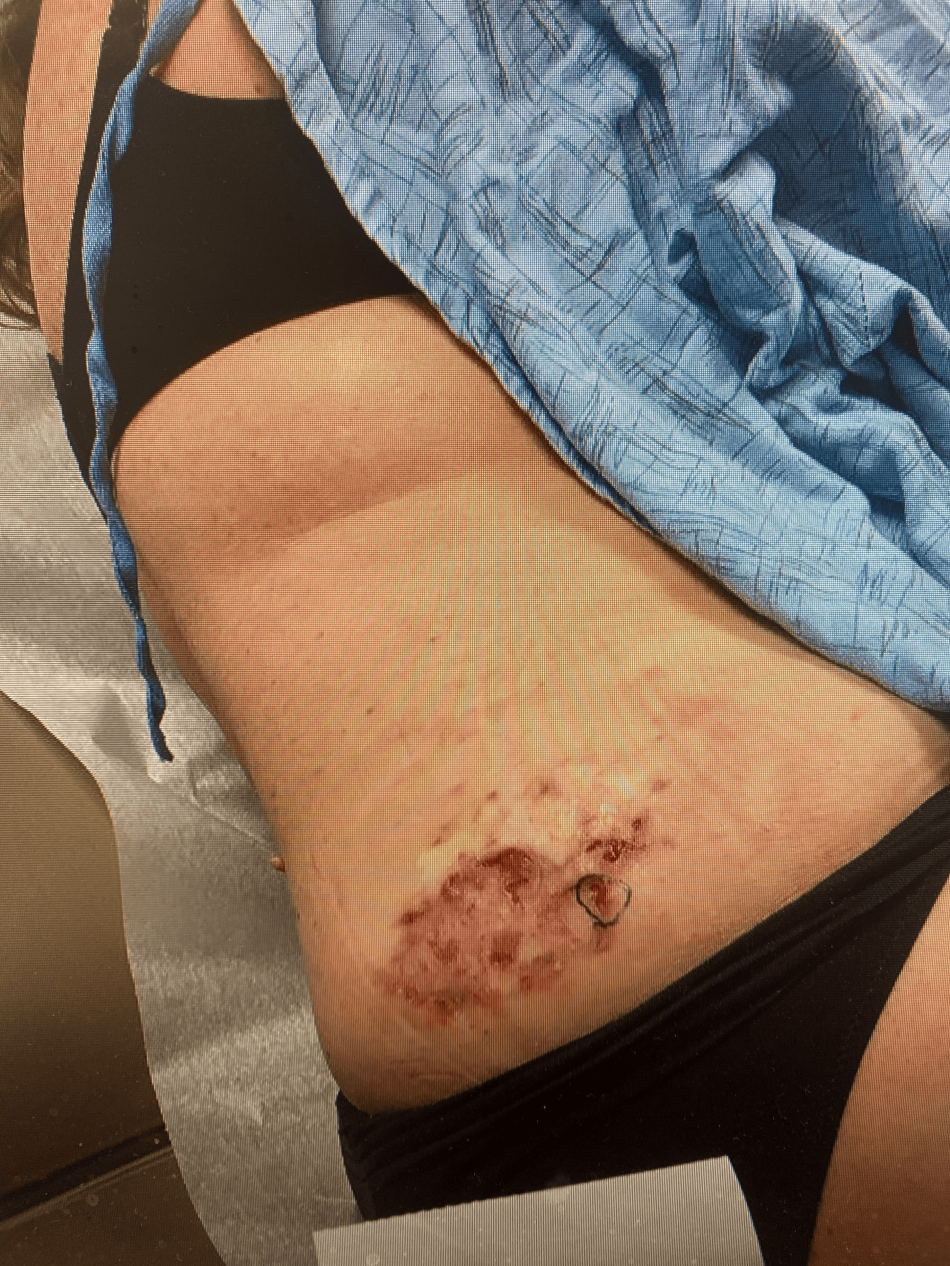
Clinical image of the right flank showing an erythematous, dark brown-to-red crusted lesion with areas of superficial erosion and ulceration. The lesion exhibited rapid growth and was located in a region prone to mechanical irritation. A surgical mark is visible.

A 57-year-old female presented to the dermatology clinic with a lesion on her right flank that she reported had been enlarging over the course of one year. On clinical examination, the lesion measured 10 cm × 6 cm and exhibited superficial erosions as well as areas of regression characterized by fibrotic or scar-like tissue (Figure 1). The patient had a previous history of melanoma but no previous history of non-melanoma skin cancer. The patient was not immunosuppressed and had no significant family history or concerns.

**Figure 2:**
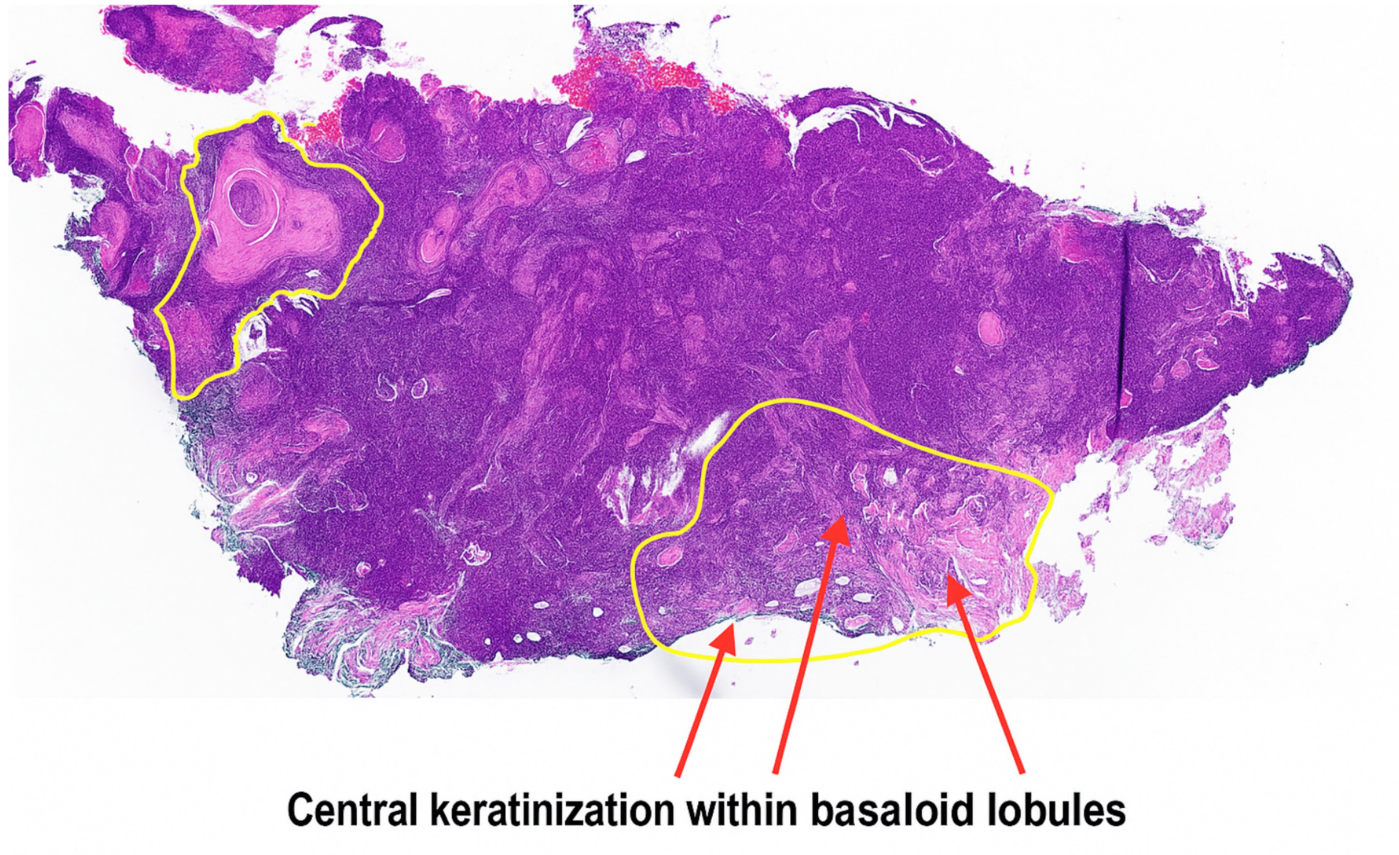
Hematoxylin and eosin stain, 4× magnification. Low-power view of a well-circumscribed dermal tumor composed of basaloid lobules with peripheral palisading and scattered areas of central keratinization. Foci of matrical differentiation are evident, consistent with BCCMD. BCCMD: basal cell carcinoma with matrical differentiation

**Figure 3:**
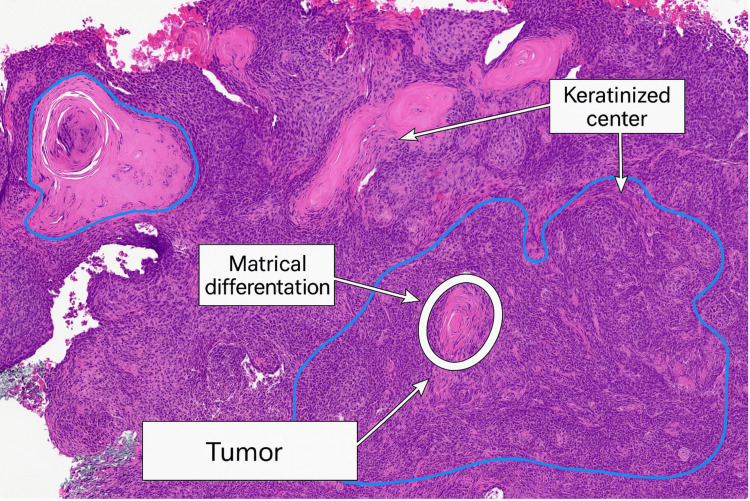
Hematoxylin and eosin stain, 10× magnification. Higher magnification shows basaloid nests with peripheral palisading and abrupt central keratinization. Matrical differentiation is demonstrated by eosinophilic keratinized centers resembling hair matrix structures, supporting the diagnosis of BCCMD. BCCMD: basal cell carcinoma with matrical differentiation

An initial punch biopsy on the right flank revealed strands and lobules of basaloid cells within the dermis, with scattered mitotic figures (Figure 2). Some lobules exhibited central keratinization reminiscent of pilar structures (Figure 3). Immunohistochemical staining with BerEP4 highlighted the lesion. These findings were consistent with a diagnosis of keratotic BCCMD.

One month later, a second biopsy from a regressed area demonstrated only scar tissue and chronic inflammation. Three months after the second biopsy, an MRI of the region was obtained to evaluate for any further spread. The MRI confirmed that the lesion was localized to the skin, with no evidence of metastasis. Surgical excision of the lesion was performed without complication approximately one month following the MRI, and the area healed with secondary intention.

## Discussion

BCC is the most common form of skin cancer [1]. It is typically characterized by slow growth, low metastatic potential, and well-defined clinical and histologic subtypes such as nodular, superficial, and infiltrative BCC. However, BCCMD represents a rare histopathologic variant, with only 42 cases reported in the literature as of 2018 [1]. The case presented here is therefore of particular significance, both because of the tumor’s rare matrical features and its unusually aggressive growth.

Histologically, BCCMD shows strands and lobules of basaloid cells in the dermis, along with scattered mitotic figures. A key feature of this variant is the presence of central keratinization within some of the lobules, which resembles the structure of early hair-forming (pilar) cells [8]. This pattern defines mathematical differentiation and helps distinguish this rare subtype from more typical forms of BCC. In this case, the tumor exhibited clear areas of central keratinization, which supported the diagnosis. Immunohistochemical staining with BerEP4 was positive, further confirming that the lesion was a BCC. This marker helps separate BCC from other tumors that may look similar under the microscope, such as pilomatricoma [8].

This tumor should be differentiated from other adnexal neoplasms, particularly pilomatricoma and pilomatrix carcinoma, which can also show matrical features. Histologically, pilomatricoma often presents with prominent shadow cells, ghost cell keratinization, and calcification but lacks peripheral palisading [8,9]. Pilomatrix carcinoma, on the other hand, tends to exhibit significant cytologic atypia, frequent mitoses, and infiltrative growth, features that are not characteristic of BCCMD [8,9]. Immunohistochemically, pilomatricoma and pilomatrix carcinoma are typically negative for BerEP4, whereas BCCMD maintains strong positivity, making this marker an important tool in distinguishing between these entities [8,9]. Furthermore, pilomatricomas and pilomatrix carcinomas frequently harbor CTNNB1 gene mutations, resulting in nuclear β-catenin accumulation, a finding not commonly observed in BCC [8,9]. These histologic and molecular differences are critical, as pilomatrix carcinoma has metastatic potential and generally requires more aggressive treatment.

While BCCMD is not typically known for its metastatic potential, there have been extremely rare cases, such as nasal BCCMD with regional metastasis and regional lymph node metastasis [5,10]. Interestingly, the review article included two cases that had a very aggressive growth pattern that resulted in metastatic spread to lymph nodes [8,11]. Our case was unusual due to the rapid growth and large size of the tumor. The lesion exhibited rapid growth over the span of one year, ultimately reaching a size of 10 cm × 6 cm. This degree of rapid enlargement is atypical for most BCCs and underscores the potential for this variant to deviate from the expected slow progression of classic subtypes. Therefore, it may indicate that a subset of patients with BCCMD may show an aggressive clinical pattern.

Table 1 shows a summary of selected cases of BCCMD reported in the literature, highlighting patient demographics, tumor site, clinical features, treatments, and outcomes.

**Table 1:** Summary of selected cases of BCCMD reported in the literature. This table highlights patient demographics, tumor site, clinical features, treatments, and outcomes. Our case, shown at the bottom, demonstrates one of the largest and most rapidly growing lesions reported to date. BCCMD: basal cell carcinoma with matrical differentiation

Study	Year	Age	Sex	Site	Clinical features	Treatment	Outcome
Kanitakis et al. [4]	2018	50	M	Face	Rapid growth; renal transplant patient	Excision	Disease-free
Haskell et al. [3]	2005	63	F	Nose	Indolent lesion	Excision	No recurrence
Kim et al. [5]	2003	43	F	Cheek	Nodular, firm	Excision	Resolved
Maroun et al. [10]	2017	72	M	Nose	Metastatic spread to lymph nodes	Excision	Treated with adjuvants
Piva de Freitas et al. [11]	2017	58	M	Scalp	Rapid growth; metastatic	Excision	Poor prognosis
Present case	2024	57	F	Right flank	Rapid growth over 1 year (10 × 6 cm)	Excision	Healing by secondary intent

## Conclusions

This case emphasizes the importance of recognizing keratotic BCCMD as a rare variant that can demonstrate unusually aggressive growth. Although BCC is typically slow-growing with low metastatic potential, this case showed rapid enlargement and a large lesion size over a short period. Accurate diagnosis, utilizing histologic features such as central keratinization and immunohistochemical staining with BerEP4, is critical. The presence of pillar structures, including shadow cells and areas of central keratinization, is key in distinguishing the matrical differentiation aspect of this variant from more typical forms of BCC. While not all BCCMD cases exhibit aggressive growth, at least two of the 42 reported cases were metastatic, and our case further supports the potential for this variant to display aggressive clinical behavior. Additionally, the trunk is a comparatively rare anatomical site for BCCMD presentation, underscoring the diagnostic and clinical significance of this case.

## References

[1] Cameron MC, Lee E, Hibler BP (2019). Basal cell carcinoma: epidemiology; pathophysiology; clinical and histological subtypes; and disease associations. J Am Acad Dermatol.

[2] Crowson AN (2006). Basal cell carcinoma: biology, morphology and clinical implications. Mod Pathol.

[3] Haskell HD, Haynes HA, McKee PH, Redston M, Granter SR, Lazar AJ (2005). Basal cell carcinoma with matrical differentiation: a case study with analysis of beta-catenin. J Cutan Pathol.

[4] Kanitakis J, Ducroux E, Hoelt P, Cahen R, Jullien D (2018). Basal-cell carcinoma with matrical differentiation: report of a new case in a renal-transplant recipient and literature review. Am J Dermatopathol.

[5] Kim SH, Lee MG, Lee KG (2003). Basal cell carcinoma with matrical differentiation. Yonsei Med J.

[6] Lam MW, Wells H, Gibbs H, Zhao A, Tso S, Wernham A (2024). P051 the what, how and why of basal cell carcinoma: natural progression and surgical intervention. Br J Dermatol.

[7] Patel PV, Pixley JN, Dibble HS, Feldman SR (2023). Recommendations for cost-conscious treatment of basal cell carcinoma. Dermatol Ther (Heidelb).

[8] Differentiation M, Basal I (1999). Basal cell carcinoma of the skin with matrical differentiation: a case report. East J Med.

[9] Pirouzmanesh A, Reinisch JF, Gonzalez-Gomez I, Smith EM, Meara JG (2003). Pilomatrixoma: a review of 346 cases. Plast Reconstr Surg.

[10] Maroun C, Alam E, Khalifeh I, Abbas O, Moukarbel RV (2017). Nasal basal cell carcinoma with matrical differentiation: risk of metastasis and impact on management. Head Neck Pathol.

[11] Piva de Freitas P, Senna CG, Tabai M, Chone CT, Altemani A (2017). Metastatic basal cell carcinoma: a rare manifestation of a common disease. Case Rep Med.

